# Particle Characterization of Washing Process Effluents by Laser Diffraction Technique

**DOI:** 10.3390/ma14247781

**Published:** 2021-12-16

**Authors:** Mirjana Čurlin, Tanja Pušić, Branka Vojnović, Nino Dimitrov

**Affiliations:** 1 Faculty of Food Technology and Biotehnology, University of Zagreb, Pierottijeva 6, 10000 Zagreb, Croatia; mcurlin@pbf.hr; 2 Faculty of Textile Technology, University of Zagreb, Prilaz baruna Filipovića 28a, 10000 Zagreb, Croatia; branka.vojnovic@ttf.unizg.hr; 3 Department for General Use Items, Croatian Institute of Public Health, Rockefeller Str. 7, 10000 Zagreb, Croatia; nino.dimitrov@hzjz.hr

**Keywords:** textiles matrix, washing, dispersion system, particles, particle size distribution, cluster analysis

## Abstract

The dominant type of polymer particles in water, sediment, and various organisms partly derives from natural and synthetic fibres released in the washing process. Pollution of aquatic recipients with these particles poses an interdisciplinary problem throughout the world. Wastewater from washing represents a dispersion system with different particle sizes that is also loaded with the source of the particles. Due to this complex system, the qualification and quantification of this type of pollution is difficult. In this paper, the laser diffraction technique was applied to characterize particles in effluents from washing and rinsing materials made of a mixture of cotton and polyester. The results obtained through the analysis prove that the laser diffraction technique is acceptable for the characterization of a composite effluent sample. The advanced statistical technique of multivariate analysis confirmed the interrelationship of the parameters of this complex dispersion system.

## 1. Introduction

The improvement of the functionality of polymers, by affecting their physical, chemical and some biological (e.g., antimicrobial) properties, can be achieved by adding chemical additives [1]. The polymer matrix and the functional additive can be connected by a covalent bond or much weaker bonds, so their release in dry and wet treatments is expected. Additional release of particles during usage, such as fibrils and fragments from polymer materials, can affect environmental systems, where the released formations can be carriers of micro-/nano-particles of substances such as dyes, pigments, functional coatings, surfactants, preparations, softeners, and others.

The issue of fibril release is related to different areas of research and testing because the assessment of fibre transfer is of particular importance for the impact of the textile industry on the environment. The process of washing textiles in the household is identified as a significant cause of environmental pollution, where the release of particles from textiles is mainly due to chemical and mechanical influences in the washing process [2,3,4,5,6,7]. The influence of chemistry is attributed to the detergent containing inorganic and organic substances of different solubility that, in synergy with other factors of the Sinner’s circle, fulfil the tasks in washing [8]. The fibrils, additives, and components of the detergent released during washing chemically and biologically load the composition of the effluent. As the material ages, the potential risk of migration of functional particles such as silver increases, which then reach the wastewater via the effluent from textile washing machines [9]. Particles released into wastewater treatment plants in this way, with an average inflow of 11 µg/L, can inhibit the growth of microbial culture, reduce the efficiency of wastewater treatment, or become toxic to aquatic organisms by streaming into natural recipients [10,11].

The influence of mechanics on the release of polymer fibrils in washing, especially when it comes from textiles, has been a focus of the scientific community in the last few years [12]. The washing machine design type affects the hydrodynamics, the transport of textiles, and the baths inside the drum. Due to resistance forces that occur inside the rotating drum and article agitation, fibrils are released and migrated, thus loading the recipients [13].

The contamination of aquatic recipients with microplastic particles originates, in part, from synthetic fibres released during the washing process; it is known that synthetic fibres are the dominant type of polyester microplastic found in water, sediment, and various organisms [14,15].

Due to the potential contamination of aquatic recipients with different particles, it is important to characterize the wastewater from washing through determining the particle size [16,17,18].

In view of the above, washing wastewater can contain a significant quantity of solid phases and form a disperse system. Such particles are classified as dissolved, colloidal, fine, and settleable solids [19]. In wastewater treatment plants, large particles are removed by standard procedures in the primary treatment process, while reducing the concentration of small particles in wastewater poses a challenge in wastewater treatment and water reuse.

Determining the volumetric or numerical particle size distribution, known as particle size analysis (PSA) or particle size distribution (PSD), is an important parameter for the physicochemical or biological wastewater treatment of drinking water, municipal and industrial wastewater, and agricultural and natural waters [20,21,22,23,24,25,26].

The present research focuses on the analysis of effluents from the washing and rinsing process by applying the PSA method, as a contribution to the characterization of released particles from cotton/PES fabric in these processes, which has not yet been applied to these phenomena. From the obtained results, the influence of process parameters on particle release was estimated using the multivariate analysis (MVA) [27].

## 2. Materials and Methods

### 2.1. Materials

**Figure 1:**
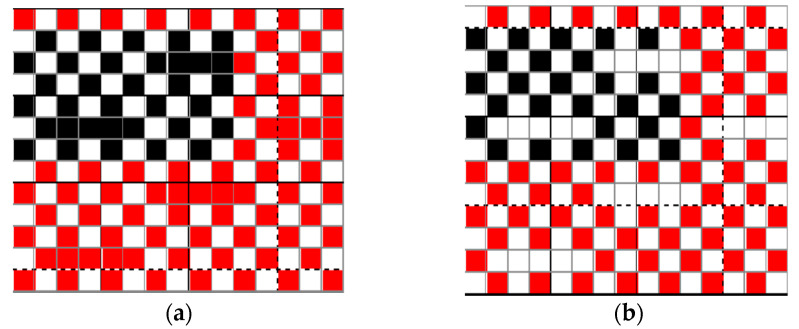
Fabric: (**a**) face side; (**b**) back side.

A fabric made of a mixture of cotton and polyester (60/40), of surface mass 270 g/m^2^, and density 37 threads in warp direction and 14 threads in weft direction was analysed. The fabric was woven in crepe weave through systematic adding of binding points in the plain weave. The finer warp and coarser weft were used. Crepe weaves produce a restless and grainy fabric appearance, Figure 1.

Samples of cotton/PES fabric were washed with water and ECE B detergent, the composition of which is shown in Table 1.

#### 2.1.1. Washing and Rinsing Procedures

Samples of fabric were washed in Polymat, W. Mathis, bath ratio (BR 1:5) at the standard programme at 90 °C with reference detergent ECE B at a concentration of 5 g/L and water through 5 cycles for 30 min.

After each cycle of washing with detergent and water, samples were rinsed in a laboratory beaker with water (BR 1:6) at 20 °C through 5 cycles. The rinsed samples were air-dried.

#### 2.1.2. Sampling

The collected composite effluents after 5 washing cycles with detergent and 25 rinsing cycles with water, as well as after 5 washing cycles with water and 25 rinsing cycles with water, were used for further analysis, as shown in Table 2.

The same samples were subjected to a membrane filtration procedure using filter from glass fibres with a pore size of 0.7 μm and a diameter of 43 mm. Filters with residual substances after drying in an oven at 105 °C (±5 °C) were tested by staining and examined by digital microscope.

### 2.2. Methods

#### 2.2.1. Turbidity

Turbidity of detergent solution and effluents (D_sample 1, D_sample 2, W_sample 1, W_sample 2) was measured by nephelometric method HRN EN ISO 7027-1: 2016. A turbidity meter Hach TL 2350 was calibrated with a certified reference material, STABLCAL (Stabilized Formazine Standard) and checked with the Gelex Secondary Turbidity Standards (GSTS).

#### 2.2.2. Particle Size Analysis (PSA)

PSA is a laser diffraction technique in which the diffraction angle depends on the particle size, i.e., the pattern of diffraction of the sample is dependent on the particle size distribution, PSD. The outcome of the measurement is a particle size distribution function [28].

The results of the analysis are described as a density or frequency distribution (*q*_3_) indicating the probability of finding a particle diameter D in the population, and a cumulative curve (*Q*_3_) indicating the percentage of particles smaller or larger than diameter D. The mathematical correlation of the cumulative and frequency curve is presented in the following Equation (1):

(1) $$Q_{3}\left(r\right)=\int_{0}^{r}q_{3}\left(r\right)dr$$

A simple graphical interpretation of the curves provides satisfactory results. The particle size distribution curve is a statistical curve, that provide important data on particle parameters in the disperse system that can be mathematically determined. These parameters include: (i) *mode*; (ii) *median* or D50, which is the value separating the higher half of the data from the lower half, which can be easily determined from the cumulative distribution curve; (iii) *mean*, which is the most characteristic value of a sample. The laser diffraction technique using volume-weighted mean diameter and calculation of these means follows the equation:

(2) $$D\left[4,3\right]=\frac{\sum n\cdot d^{4}}{\sum n\cdot d^{3}}$$

Apart from the above statistical parameters, the most common values used in particle sizing technique are the D-values, which means that generally D_x_ presents the diameter, of which an x percentage of the particles is smaller. The three most frequently used D-values are D_10_, D_50_, and D_90_, but custom D-values can be found for the characterization of samples.

A helpful description of the particle size distribution usually requires a method of describing the shape of the distribution. An important measure of any statistical distribution is the width or broadness. A frequently used measure in laser diffraction is the span. In general, it is calculated by the following equation:

(3) $$\mathrm{Span}=\frac{D_{90}-D_{10}}{D_{50}}$$

The Particle Size Analysis (PSA) was performed according to a standard measurement procedure for each sample in a series of three repetitions on a PSA 1190 LD Anton Paar GmbH, Austria. The optimization of input parameters such as stirring and pump optimization allows one to obtain consistent and repeatable results. As a dispersion medium, water was used, and obscuration was 9–13%; stirrer and pump speed were medium.

#### 2.2.3. Cluster Analysis

Cluster analysis using TIBCO Statistics software was applied to evaluate different parameters of washing and rinsing impact on particles in effluents. This method is part of multivariate statistical analysis (MVA), which is used to analyze complex data sets and their relationships. We used this empirical method, which places similar objects that are close to each other in the space of variables into the same class, for the particle size distribution data of washing and rinsing treatments. The result is presented in the form of a dendogram of the stepwise combination of objects in clusters. The Euclidean distance defined by Equation (4) is the distance d between two points in n-dimensional space with coordinates *x_j_* and *y_j_* [27].

(4) $$d_{x,y}=\sqrt{\overset{J}{\underset{j=1}{\sum }}{\left(x_{j}-y_{j}\right)}^{2}}$$

#### 2.2.4. Characterisation of Filter Residues

The solid residue on the filter was stained by soaking in cold solution of 2% Bezaktiv Brilliant Blau V-R spez. (C.I. Reactive Blue 19) from Bezema for 15 min. Afterwards, filters were dried and examined with the digital microscope Dino-Lite Premier, IDCP under magnification of 50× and 230×.

## 3. Results and Discussion

The source of solid particles in washing effluents can be textile material, detergent, or water, which makes it difficult to choose methods for their characterization. This research focused on the application of PSA in the analysis of effluents collected as composite samples from five washing cycles and 25 rinsing cycles of a cotton/PES fabric. The first effluent is from washing with standard detergent (D_sample 1), the second is from subsequent rinsing with water (D_sample 2), the third is from washing with water (W_sample 1), and the fourth is from subsequent rinsing with water (W_sample 2), Table 2.

Results of turbidimetric analysis of detergent solution and effluents are presented in Table 3.

The analysis of turbidity results in Table 3 shows the difference between washing and rinsing effluents. The turbidity value of detergent solution is 255.7 NTU and correlates with the composition shown in Table 1. The turbidity of effluent D_sample 2 is three times lower than that of D_sample 1, while the turbidity of W_sample 2 is four times higher than that of W_sample 1. The increase in turbidity may be influenced by fibril fragments. This hypothesis was underlined by the laser diffraction method as follows.

PSA was applied as the overlay of three consecutive measurements for the density distribution and the cumulative distribution as displayed in figures and tables. This is a common way of showing the particle size distribution where the function is obtained by applying Equation (1).

**Figure 2:**
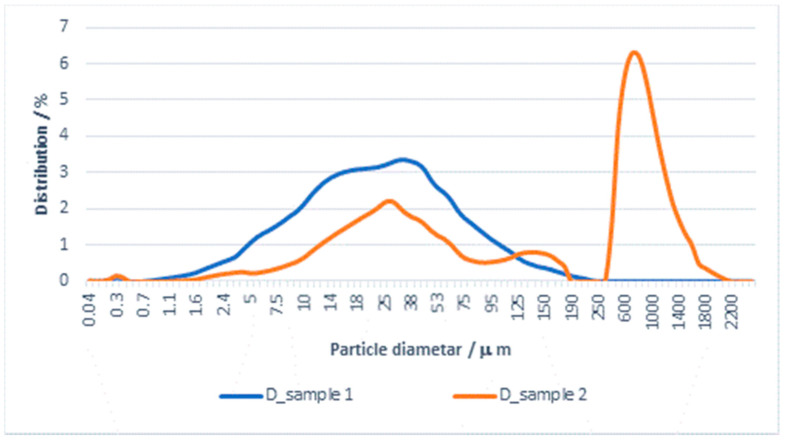
Distribution of particles (PSA) in effluent from washing a cotton/PES fabric with detergent and subsequent rinsing with water.

**Figure 3:**
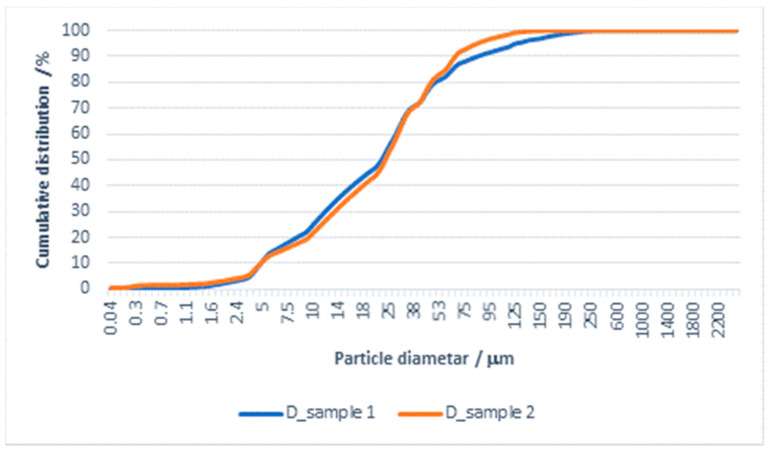
Cumulative distribution of particles (PSA) in effluent from washing a cotton/PES fabric with detergent and subsequent rinsing with water.

The results of the particle size distribution for the effluent from washing in detergent and subsequent rinsing are shown in Figure 2 and Figure 3.

Based on the results of effluent analysis, the D-value was determined for particles smaller than 10, 50, and 90 µm. The results for mean values of D_10_, D_50_, D_90_, and Span which determine according to Equation (3) are shown in Table 4.

From the PSA results shown in Table 4 and Figure 2 and Figure 3 for effluents from washing in detergent and subsequent rinsing, it can be seen that both samples belong to monodisperse systems, with the mean particle size and D_90_ being smaller for the effluent from rinsing. A comparison of Span values calculated according to Equation (3) for both samples indicate differences in particle distribution by diameter. The Span value for the effluent from washing is 3657, whereas for the effluent from rinsing it is 2661. In this system, the detergent has a significant effect on the effluent load with particles, despite the fact that the rinsing process was carried out in water, i.e., without detergent.

The role of detergent in this “clean” system could not be for removing the soil, and it tends to load the material surface and, partly, the effluent. Therefore, the rinsing process removes most of the residual substances from the material, which puts a load on the effluent.

**Figure 4:**
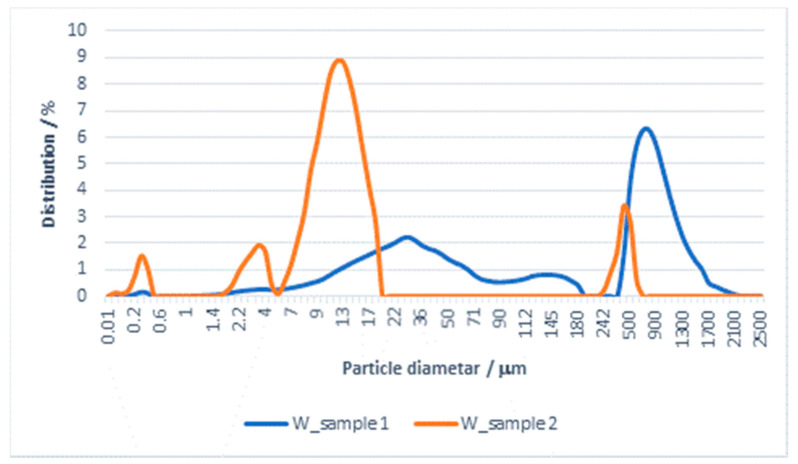
Distribution of particles (PSA) in the effluent from washing a cotton/PES fabric with water and subsequent rinsing.

**Figure 5:**
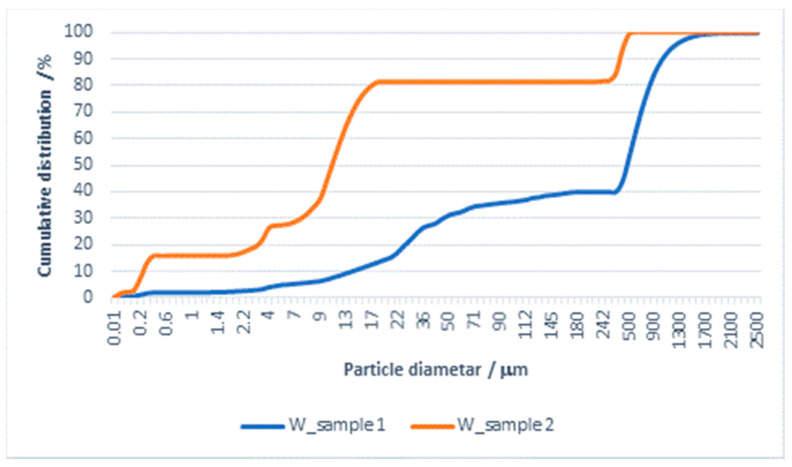
Cumulative distribution of particles (PSA) in the effluent from washing a cotton/PES fabric with water and subsequent rinsing.

The results of the particle size distribution for the effluent from washing in water and subsequent rinsing are shown in Figure 4 and Figure 5 and Table 5.

The PSA results for effluents from washing with water and subsequent rinsing show that both samples belong to polydisperse systems, with the mean particle size and all the presented diameters being significantly smaller for the samples of water from rinsing.

By comparing these values with the values obtained for the effluent from washing with detergent, significant differences can be observed. There is also a major difference in the mean value of the particles (mean size) in the effluent from washing in water (482.899 µm) in relation to the effluent from rinsing, where it is 77.033 µm. The Span values, calculated according to Equation (3) for both samples, indicate differences in the distribution of particles by diameter; for the effluent from washing it is 2243, whereas for the effluent from rinsing it is 33.467. The migration of particles in the processes of washing and rinsing in water can, in a certain way, be associated with the material surface and, in these effluents, can be characterized as particles of textile origin.

The factors of the Sinner’s circle in the observed disperse washing and rinsing systems are different. In the process of washing the fabric with detergent, the chemical effect takes place through the action of its components (Table 1) in synergy with the mechanics through the reverse direction of rotation 60/60 with 40 RPM in a bath at 90 °C for 30 min.

The process of washing the fabric with water takes place with the same mechanics, temperature and time, while the interaction of the bath with the material takes place in water, without chemical effects of detergent. It is known that in such detergent-free washing systems the influence of mechanics, time, and temperature is dominant [7]. The indicators of the distribution of released particles show that higher temperature and mechanical agitation in the washing process contributed to a more significant release of particles.

After the washing process in detergent and water, the rinsing process was carried out through five cycles in a laboratory beaker, by gently stirring the sample in water at 20 °C for 3 min. Thus, the results of the particle diameter analysis indicate the migration of particles completely from the cotton/PES fabric into the effluent.

**Figure 6:**
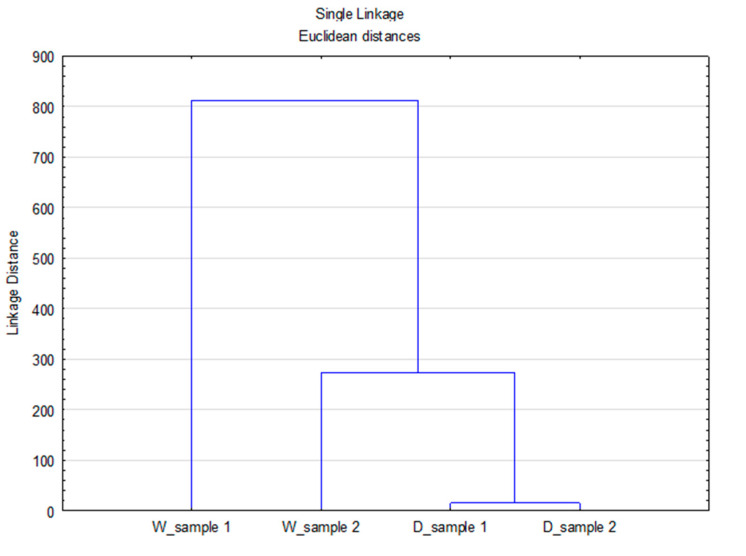
Dendogram of the CA with Linkage Distances of all data sets of volume distribution particles.

The importance of the Sinner’s circle parameters [7] in the washing process and their interrelationship implies the need for conducting more advanced analyses to better define their influence on the degree of effluent load. In view of the observed disperse system in which particles are from different sources (detergent and material) and different geometric forms (particles and fibrils), it is important to group the parameters and define their interrelationship in the process of washing and rinsing. To obtain important additional information about the observed system, cluster analysis was performed. To perform these analyses, the data of the volume distribution were used, Figure 6.

The obtained Linkage Distance values indicate the differences between the observed systems through the separation of one effluent (W_sample 1) into a separate group from the other effluents. The potentially released particles in this effluent originate exclusively from the previously highlighted Sinner’s circle parameters (temperature, mechanics, and time) and can be geometrically characterized as fibrils. In the next group there is the effluent from rinsing (W_sample 2) followed by a group consisting of two effluents from washing with detergent (D_sample 1) and rinsing (D_sample 2). The presented Linkage Distance for these groups indicates some smaller differences in effluents, which can be attributed to the composition of the bath, dominated by detergent particles with a small proportion of particles with fibril geometry, which was confirmed by separating effluent W_sample 2 into a separate group.

**Figure 7:**
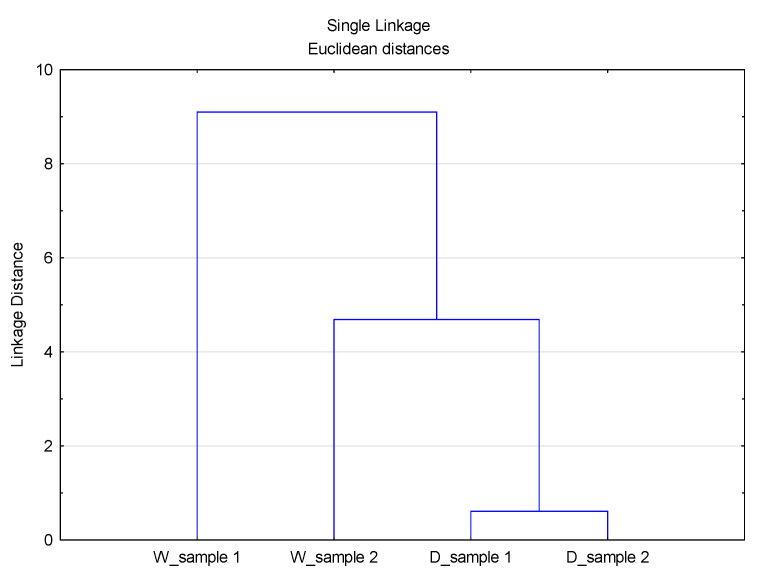
Dendogram of the CA with Linkage Distances of all data distribution sets for diameter D_10_.

**Figure 8:**
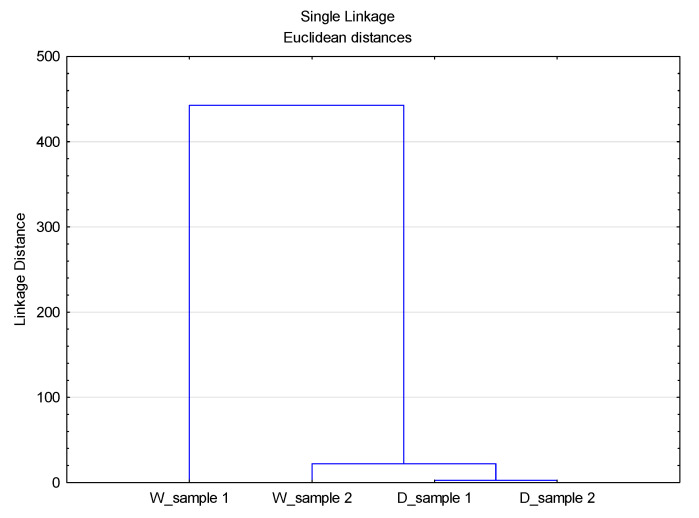
Dendogram of the CA with Linkage Distances of all data distribution sets for diameter D_50_.

**Figure 9:**
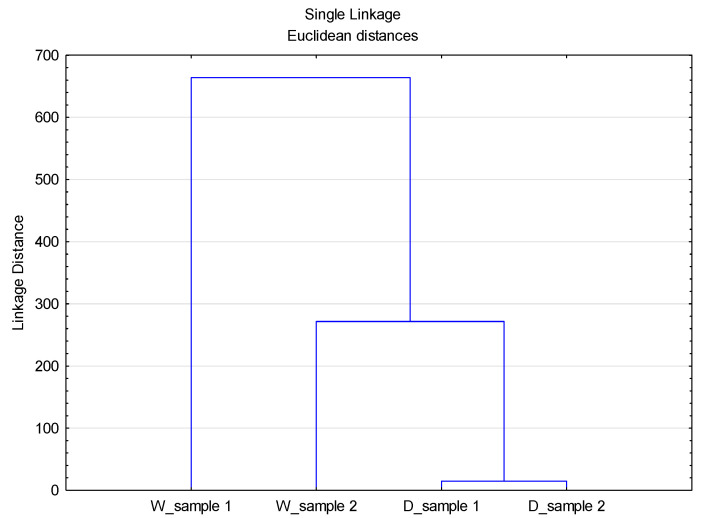
Dendogram of the CA with Linkage Distances of all data distribution sets for diameter D_90_.

A cluster analysis of the data sets of volume, surface, and number distributions for different D values was performed, Figure 7, Figure 8 and Figure 9.

As can be seen from Figure 7, Figure 8 and Figure 9, the previously stated thesis about the source of particles in the observed system is confirmed. The Linkage Distance of the observed effluents is significantly smaller for particles of smaller diameter (D_10_) compared to diameter D_50_ and diameter D_90_.

**Figure 10:**
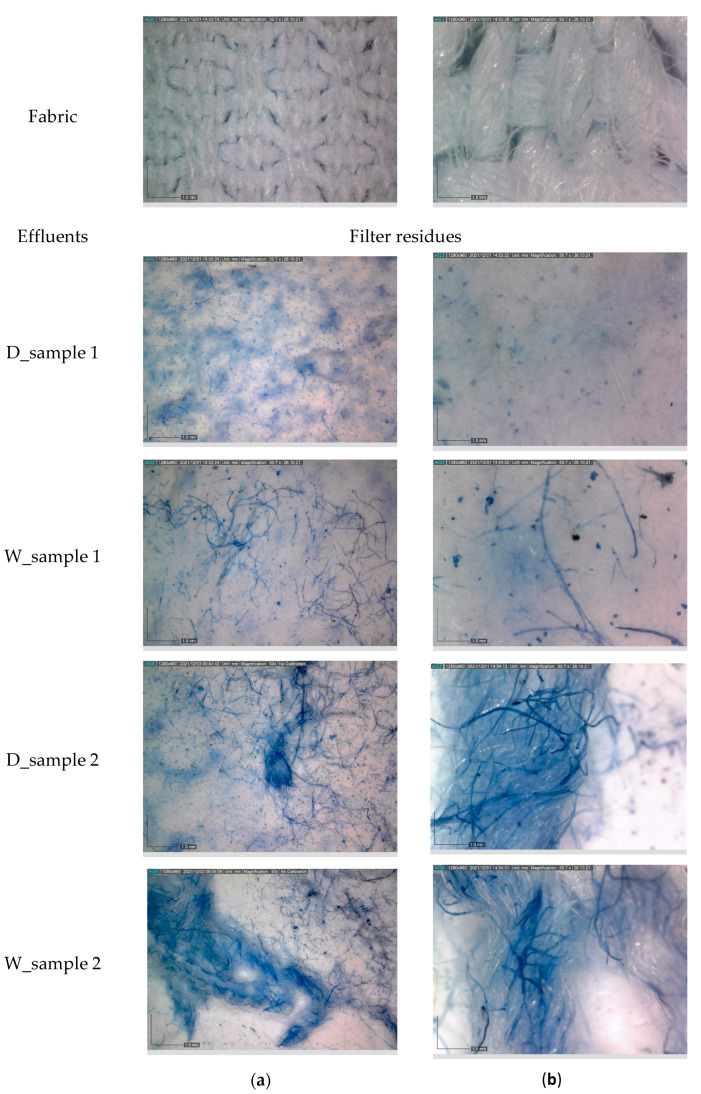
Digital images of stained and non-stained solid residues: (**a**) magnified 50×; (**b**) magnified 230×.

The examination of fabric and solid residues on filter after staining by digital microscope is shown in Figure 10.

The digital images of particular sections of the filter magnified 50× and 230× in Figure 10 show the different compositions of the residues. The roughness in the filter surface of D_sample 1 and D_sample 2 is affected by detergent residues with fibrillar fragments integrated into the matter contents. The comparison of the images of D_sample 2 and D_sample 1 shows a low rough filter surface of D_sample 2. At the same time, the fibril content in D_sample 2 is more obvious than in D_sample 1.

The filter content in W_sample 1 and W_sample 2 is mostly covered by fibrils, whereas the W_sample 2 is characterized by grouped fibrils in comparison to the W_sample 1.

The fabric in crepe weave was made from fine warp threads of cotton with polyester and the coarse weft threads just from cotton. The technical characteristics of the threads caused specific distribution of cotton and polyester fibrils as filter content, especially in D_sample 2 and W_sample 2, where cotton fibrils are blue stained. Polyester fibrils retained a pristine colorless form.

## 4. Conclusions

The results of the analysis of effluents from the washing and rinsing process of cotton/PES fabrics by the laser diffraction method contribute to the characterization of migrating and released particles, and point to the importance of observing their source and specific geometry. The specific geometry of the released particles, observed in the digital images, is due to the structure and composition of the fabric as well as the action of the detergent and the parameters of the washing and rinsing process. A complex dispersion system consisting of material, detergent, water, and the influence of Sinner’s circle parameters requires the application of advanced statistical analyses. The results of the analysis provided an additional understanding of the interrelationships in the observed complex system, especially through the classification into groups based on process phases and characteristic diameters.

## Figures and Tables

**Table 1:** Composition of reference detergent ECE B.

Ingredient	w (%)
Linear sodium alkylbenzene sulfonate (LAS)	8.0
Ethoxylated fatty alcohol C12/18 (14EO)	2.9
Sodium soap (C 12−16: 13−26%. C 18−22: 74−87%)	3.5
Sodium triphosphate	43.7
Sodium silicate (SiO_2_:Na_2_O = 3.3:1)	7.5
Potassium silicate	1.9
Carboxymethylcellulose (CMC)	1.2
Ethylenediaminetetraacetic acid	0.2
Sodium sulphate	21.2
Water	9.9
Σ	100

**Table 2:** Designation of effluents.

Washing	Effluent 1	Rinsing	Effluent 2
ECE B detergent	D_sample 1	Water	D_sample 2
Water	W_sample 1	Water	W_sample 2

**Table 3:** Turbidity of detergent solution and effluents.

Sample	Turbidity/NTU
Detergent solution	255.7
D_sample 1	64.2
W_sample 1	2.20
D_sample 2	20.4
W_sample 2	8.85

**Table 4:** Results of volume-weighted D-value for effluents from washing with detergent and rinsing.

Mean Value/µm	D_10_/µm	D_50_/µm	D_90_/µm	Mean Size/µm	Span
D_sample 1	4.726	21.830	84.549	37.116	3.657
D_sample 2	4.935	23.968	68.710	33.027	2.661

**Table 5:** Results of volume-weighted D-value for effluents from washing in water and rinsing.

Mean Value/µm	D_10_/µm	D_50_/µm	D_90_/µm	Mean Size/µm	Span
W_sample 1	14.726	439.013	998.805	482.899	2.243
W_sample 2	0.2378	10.527	352.498	77.033	33.467

## Data Availability

The data are open available.
